# A Retrospective Chart Review Evaluating Changes in 25-Hydroxyvitamin D Levels among Patients Attending the University Healthcare Centre during the COVID-19 Pandemic

**DOI:** 10.3390/nu15102345

**Published:** 2023-05-17

**Authors:** Tarek Benameur, Feroze Kaliyadan, Neji Saidi, Chiara Porro

**Affiliations:** 1 Department of Biomedical Sciences, College of Medicine, King Faisal University, Al Ahsa 31982, Saudi Arabia; 2 Sree Narayana Institute of Medical Sciences, Kerala University of Health Sciences, Thrissur 683594, India; 3 Department of Mathematics and Statistics, College of Sciences, King Faisal University, Al Ahsa 31982, Saudi Arabia; 4 Department of Clinical and Experimental Medicine, University of Foggia, 71121 Foggia, Italy

**Keywords:** 25-hydroxyvitamin D, vitamin D, COVID-19, deficiency, insufficiency, lockdown, pre-lockdown, post-lockdown, Saudi Arabia, pandemic, home confinement, coronavirus disease

## Abstract

COVID-19-pandemic-related home confinement aids in limiting the spread of the virus but restricts exposure to sunlight, thereby possibly affecting 25(OH)D concentrations. This study aimed to investigate the effect of lockdown measures on 25(OH)D levels in outpatients visiting the healthcare centre over a period of two years. In this retrospective chart review, outpatients who visited a university healthcare centre for a health check-up over a period of two years were included. The patients’ 25(OH)D serum levels and status were compared before, during, and after the lockdown periods. A total of 7234 patients were included in this study, with a mean age of 34.66 ± 16.78. The overall prevalence of 25(OH)D insufficiency, deficiency and sufficiency was 33.8%, 30.7% and 35.4%, respectively. The proportion of individuals with 25-(OH) D deficiency prior to lockdown was 29% and this proportion increased in the lockdown and post-lockdown periods to 31.1% and 32%, respectively. Although gender was less likely to have an impact on the 25 (OH) D level during the lockdown period (*p* = 0.630), we found an association between gender and 25 (OH) D status in the pre-lockdown and post-lockdown periods (*p* < 0.001 and *p* < 0.001, respectively). Another association between nationality and 25 (OH)D levels was found before, during and after the lockdown periods (*p* < 0.001). In addition, the youngest population, aged between 1 and 14, was strongly affected by the home confinement. Age had a positive and significant (*p* < 0.05) effect on 25 (OH) D status regardless of the different periods. Moreover, in the pre-lockdown period, male outpatients had 1.56 chance of having a sufficient level of 25 (OH)D. However, during the lockdown period, this chance decreased to 0.85 and then increased to 0.99 after the lockdown period. We found no statistically significant difference in the mean serum concentrations or in the prevalence of vitamin D insufficiency when we compared values from before, during and immediately after the COVID-19 lockdown period. However, there was a generally increased prevalence of vitamin D insufficiency in our study population. Another association between gender, nationality and age groups with 25(OH) D was found. Regular exposure to UVR is recommended for maintaining adequate vitamin D levels and to prevent vitamin D deficiency. Further research is needed to determine the best indications for vitamin D supplementation if confinement periods are extended and to consider the potential health consequences of prolonged confinement periods not only on vitamin D status but also on overall public health. The findings of this study may be considered by stakeholders for a targeted supplementation approach for risk groups.

## 1. Introduction

Vitamin D (1,25 dihydroxyvitamin D3) is produced in the skin in response to exposure to *UVB* radiation and can be obtained to a lesser extent from dietary sources [1,2]. Vitamin D plays a critical role in maintaining bone density, building the immune system and regulating cell growth, among other key biological functions. 

In recent years, the frequency of vitamin D testing has exponentially increased in primary health care [3]. According to Endocrine Society guidelines, serum 25-hydroxy-vitamin D: 25(OH)D < 20 ng/mL is indicative of a deficiency, levels between 21 and 29 ng/mL are classed as “insufficient” and recommended normal levels correspond to a circulating level ≥30 ng/mL [4,5,6]. Although vitamin D supplementation is highly recommended from childhood to adulthood [7,8], observational studies have shown that 25(OH)D circulating levels tend to be higher among the younger age group and then decline in older age [9]. This is mostly due to physiological, social and lifestyle alterations [10]. A variety of factors influence vitamin D levels, including health status, supplement use, season and environmental factors such as air pollution, age-related behavioural changes, such as clothing and outdoor time, and age-related changes in vitamin D metabolism [11,12]. Vitamin D deficiency is a worldwide health problem that primarily affects musculoskeletal health but also a wide range of acute and chronic diseases, ranging from hair loss to cardiovascular disease, bone disease, cancer, diabetes, infertility, multiple sclerosis, respiratory tract infections such as coronavirus disease (COVID-19) and many other conditions [13,14,15,16,17]. 

COVID-19, an acute respiratory infection that initially emerged in China and is caused by SARS-CoV-2, developed into a global pandemic [18]. The pandemic has altered many aspects of lifestyle behaviours, including physical activity, decreased outdoor activities, altered sleep, social distancing, prolonged isolation and dietary habits, which have led to weight gain and other conditions during the lockdown period [19,20,21,22]. Recent large observational studies have reported varying prevalence rates of vitamin D deficiency, including 37% (Canada), 24% (USA), and 40% (Europe). Comparably high prevalence rates of low vitamin D levels were reported in Tunisia, India, Afghanistan, and Pakistan [9,23,24,25,26]. In Saudi Arabia, the prevalence of vitamin D deficiency affects 60% of the population [27]. Vitamin D deficiency is more common in females and in the younger age groups [28,29]. Age, gender, traditional clothing, deliberate avoidance of sun exposure, inadequate dietary intake, the hot weather and decreased outdoor activities were all reported as risk factors for vitamin D low levels [30]. 

Additionally, the strict lockdown and consequent home confinement imposed by governments worldwide, including in Saudi Arabia, has resulted in less time spent outdoors and possibly less exposure to the sunlight that is required to maintain an adequate level of vitamin D. We postulated that it could also diminish the daily amount of vitamin D when associated with changes in eating habits, with most meals ordered through food delivery joints, which have lower nutritional and vitamin D contents [31]. 

Taken together, we postulate that this could lead to general decline in vitamin D status in the general population and might indirectly lead to an increased incidence of medical problems associated directly or indirectly with vitamin D deficiency [32]. However, data on the association between lockdown measures and vitamin D status in patients in Saudi Arabia are lacking. Hence, the aim of this study was to investigate whether the COVID-19 pandemic lockdown period, with its approach to home confinement measures, had an impact on vitamin D concentrations among the outpatients visiting polyclinics. 

This study would help to validate this hypothesis and could open the path for relevant public health measures to address this concern.

## 2. Materials and Methods

### 2.1. Study Design and Settings

This retrospective chart review was carried out to investigate the records of patients attending the outpatient department of the University healthcare centre at King Faisal University, located in the city of Al-Ahsa, Saudi Arabia, between 1 September 2019 and 24 February 2021. 

### 2.2. Participants

The data of 7234 outpatients were extracted from the electronic health records system of the University polyclinics. The study included all patients of either gender who had their vitamin D levels tested at least once within 7 months before the lockdown, during the lockdown and after the lockdown period as per the distributed periods below which were implemented by the concerned authority, as previously described in [33,34,35].

Period 1: from 1 September 2019 to 8 March 2020 (pre-COVID-19 nationwide lockdown); period 2: from 09 March 2020 to 20 June 2020 (COVID-19 nationwide lockdown); period 3: from 21 June 2020 to 24 February 2021 (post-COVID-19 nationwide lockdown) 

### 2.3. Inclusion and Exclusion Criteria

Irrespective of age, the records of all outpatients who had their 25 (OH) D levels tested at least once during the selected periods and who had completed relevant demographic data entered in their electronic medical record were included. The exclusion criteria concerned only those with incomplete demographic data.

### 2.4. Biochemical Analysis

Blood samples were collected from outpatients who visited the healthcare centre, and serum 25(OH) D levels were measured via a radioimmunoassay technique (Roche Diagnostics, Mannheim, Germany). Due to its relatively long half-life, serum 25 (OH)D concentrations, rather than 1, 25 (OH)2D concentrations, were the primary recognized indicator of vitamin D status, representing the endogenously produced vitamin D as well as vitamin D received from diet and supplementation [8,36]. 

### 2.5. Statistical Analysis 

The extracted data were analysed using an SPSS 26.0 software package, IBM SPSS Statistics for Windows (IBM Corp., Armonk, NY, USA). The serum 25(OH)D levels are presented as means and standard deviations (±SD), and categorical variables are presented as absolute values and relative frequencies. 

Mean serum 25(OH)D levels were compared between different pandemic periods (September 2019 to February 2021), using a one-way analysis of variance (ANOVA), and Pearson’s chi-square test was used to test the significance of the difference in vitamin D levels between more than two groups and two groups, respectively. Factors affecting vitamin D were determined using a linear regression analysis. A correspondence analysis was performed for certain variables including gender, age, nationality, and the visit date. In addition, a linear regression analysis was used to analyse the relationship between vitamin D levels and socio-demographic characteristics, including age, gender, and nationality, within the three previously mentioned pandemic periods. 

## 3. Results

### 3.1. Patients’ Characteristics and Their Overall 25(OH) Vitamin D Status 

Table 1 shows the characteristics of the patients. A total of 7234 patients, with ages ranging from 1 to 105 years, were included in this study. The mean age of the studied patients was 34.66 ± 16.78 (median age of 33). Among the studied individuals, 5302 were female (73.3%), and 1932 were male (26.7%). Most of the outpatients were Saudi (5461, 75.5%), and 1773 (26.7%) were non-Saudi patients. The mean serum vitamin 25(OH) concentration of the total study population was 27.7 ± 13.0 ng/mL for all periods. The overall prevalence (total population) of 25 (OH) vitamin D insufficiency, deficiency and sufficiency was 33.8%, 30.7% and 35.4%, respectively. 

### 3.2. The 25-(OH) D Serum Level during COVID-19 Pre-Lockdown, Lockdown and Post-Lockdown Periods 

To investigate whether the strict lockdown directives implemented by the Saudi authorities had an impact on the 25-(OH) D concentrations in patients visiting the University primary healthcare centres, we first compared the serum 25-(OH)D levels in the pre-lockdown, during lockdown and post-lockdown periods. The average serum 25-(OH) D concentrations recorded in these periods were 27.6, 27.4 and 27.8 ng/mL, respectively. Even though no significant difference in the average serum concentrations between groups was recorded in these periods (*p* = 0.729), this confirms an insufficient status among the outpatients (Table 2).

As illustrated in Table 2, the outpatients were classified into three groups based on their 25(OH)D concentrations as sufficient ≥30 ng/mL, insufficient (21–29 ng/mL) and deficient (0–20 ng/mL). The proportion of individuals with 25-(OH) D deficiency in the period prior to the lockdown was 29%, and this proportion increased in the lockdown and post-lockdown periods to 31.1% and 32%, respectively. Interestingly, the overall prevalence of vitamin D insufficiency was relatively high among the outpatients prior to the lockdown period (37.5%) when compared to the lockdown and post-lockdown periods (34.2% and 31.3%, respectively). Remarkably, we found that there is an association between the outpatients’ circulating levels of 25 (OH) D and the three periods (*p* < 0.001). However, there were no significant differences between the patients’ mean concentrations within the three home confinement periods (*p* = 0.729). 

When we compared the proportion of outpatients who were at risk of developing health problems due to 25 (OH) D insufficiency and deficiency combined during the pre- and post-lockdown periods, we found that this risk increased by 10% from 26.5% (1910) to 36.3% (2619).

#### 3.2.1. Changes in Serum Levels of 25 (OH) Vitamin D Stratified by Gender

As depicted in Table 3, during the period preceding the lockdown restrictions, the prevalence of 25 (OH) D deficiency was significantly higher in female patients (24.7%) compared to male patients (4.3%). Similarly, during the post-lockdown period, a significant difference in the prevalence of 25 (OH) D deficiency was also found (23.7% and 8.2%). However, during the lockdown period, there was no significant difference in the prevalence of 25 (OH) D deficiency between male and female patients (17.3% and 13.8%) among the total population. Our analysis illustrated that gender was less likely to affect the 25 (OH) vitamin D level during the lockdown period (*p* = 0.630). The comparison of the prevalence of 25 (OH) vitamin D deficiency and insufficiency between males and females regardless of the pandemic period showed a significant difference between males and females ((6.8%; 23.9%) and (9.7%; 24.1%), respectively). Table 3 shows an association between gender and the vitamin D status in the pre-lockdown and post-lockdown periods (*p* < 0.001, *p* < 0.001), respectively.

#### 3.2.2. 25 (OH) Vitamin D Status Stratified by Nationality

As shown in Table 4, the comparison of the mean concentrations of 25 (OH) D between Saudi and non-Saudi outpatients showed a significant difference between Saudi and non-Saudi patients in the pre-lockdown and post-lockdown periods (*p* < 0.001, *p* < 0.001), respectively. It should be noted that the 25 (OH) D mean concentration of Saudi patients belonged to the insufficient status during all periods, while non-Saudi patients showed a sufficient level only in the pre-lockdown period.

As demonstrated in Table 5, the prevalence of 25 (OH) D deficiency was significantly higher in Saudi outpatients when compared to non-Saudi outpatients for all periods (25.3% vs. 3.7%; 21.4% vs. 9.7%; and 26.2% vs. 5.7%, respectively). Similarly, the proportion of Saudi patients with 25 (OH) D insufficiency was higher in Saudi compared to non-Saudi patients during the three periods (pre-lockdown, lockdown and post-lockdown) (28.6% vs. 8.8%; 21.4% vs. 12.8%; 22.10 vs. 9.2).

Our data shows an association between nationality and vitamin D concentration before, during and after the lockdown restriction periods (*p* < 0.001, *p* < 0.001, and *p* < 0.001).

### 3.3. 25(OH) Vitamin D Status of Different Age Categories within the Three Periods

Table 6 summarizes the mean of 25 (OH) D concentrations among the various age categories within the pre-lockdown, lockdown and post-lockdown periods. We noticed a significant difference between the means of various age groups in the pre-lockdown and post-lockdown periods (*p* < 0.001 and *p* < 0.001, respectively). However, there was no significant difference between the means of the 25 (OH) D concentrations of the various age groups during the lockdown period.

**Figure 1:**
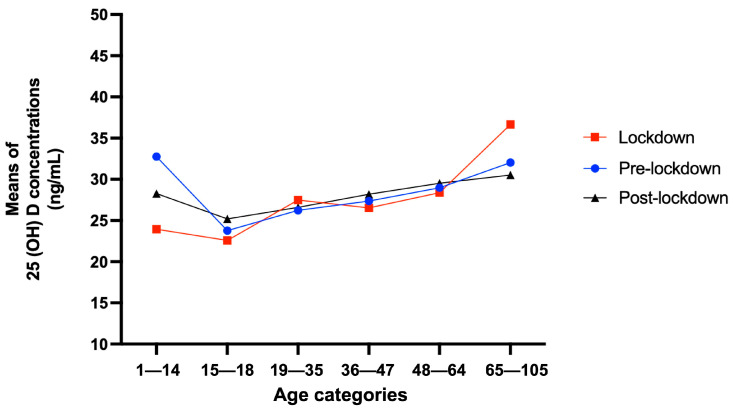
Mean of 25 (OH) D concentrations among age groups before, during and after home confinement restrictions.

As presented in Figure 1, the youngest population, aged between 1 and 14, was affected by the strict lockdown measures; this is reflected in the insufficient status of 25 (OH) D observed in the lockdown and post-lockdown periods: 23.9 ng/mL and 28.2 ng/mL, respectively. Intriguingly, the older population (65-105) was less likely to be affected by these strict directives, and all means of 25 (OH) D concentrations for this category were above 30 ng/mL during all periods, indicating sufficient levels.

The Chi-square test was used to analyse the relationship between age categories and 25 (OH) D status within the three above-mentioned periods. Interestingly, we observed that before implementing the strict lockdown and during the post-lockdown periods there was an association between 25 (OH) D status and the various age categories. However, during the lockdown period, 25 (OH) vitamin D changed independently of the age categories. This confirms that there was no association between these two variables during this period.

Table 7 shows the linear regression analysis used to estimate the impact of age, gender and nationality on the circulating levels of 25 (OH) D in outpatients during the three above-mentioned periods. Our regression analysis shows that age affected the serum 25 (OH) vitamin D level positively and significantly (*p* < 0.05) regardless of the period. However, being of Saudi nationality affected 25 (OH) vitamin D serum level negatively and significantly during only two periods: the pre-lockdown and post-lockdown periods. 

In addition, being male had a significant, positive impact on the serum 25 (OH) D level before lockdown measures were implemented (*p* < 0.001), but this impact disappeared during the lockdown and post-lockdown periods.

In conclusion, our analysis confirmed what we supposed earlier: that gender and nationality are less likely to affect the serum level of 25 (OH) D during lockdown.

Our analysis confirmed the absence of a link between gender and nationality (as described above in results section, see Table 3 and Table 4) with the vitamin D serum level during the lockdown period. As illustrated in Table 8, the binary logistic regression analysis (odd ratio = 1.56) shows that in the pre-lockdown period, the male outpatients had a 1.56 chance to be sufficient (without any problem in vitamin D level). However, during the lockdown period, this chance decreased to less than 1 (0.850) and then began to increase to almost 1 (0.997) during the post-lockdown period. 

## 4. Discussion

To the best of our knowledge, this study is the first of its kind to explore changes in 25(OH)D serum levels in outpatients in Saudi Arabia before, during and after the COVID-19-pandemic-related home confinement. Herein, we have provided novel insight into the impact of the nationwide lockdown on the 25 (OH) D serum levels of outpatients visiting the primary healthcare polyclinic, which were measured in the laboratory of the health care centre. We have compared the concentration of 25 (OH) D in the outpatients’ sera during the period preceding the nationwide lockdown with the lockdown and post-lockdown periods. We have also investigated the association of predictors of changes in 25 (OH) D levels such as gender, nationality and age.

The nationwide lockdown was implemented by the Saudi authorities to curb COVID-19 deaths, stem the spread of SARS-CoV-2 and prevent the healthcare systems from collapsing. While the home confinement measures, social distancing and the cessation of outdoor activities have slowed the transmission of SARS-CoV-2, such measures might not only impact lifestyle but may also have unintended consequences on human mental and physical health, including metabolism, with particular focus on vitamin D production secondary to sun exposure [32,37,38,39,40]. The present retrospective chart review supports this hypothesis.

The major findings of this study showed: (i) the average serum concentrations of 25-(OH) D for the study population within the three periods showed an insufficient level. (ii) We identified an increase of 10% in members of the population who are at risk of developing health problems due to both vitamin D insufficiency and deficiency when we compared the pre-lockdown and post-lockdown periods. (iii) There is an association between the 25 (OH) D level and the home confinement restrictions, (iv) and another association between gender and 25 (OH) D serum level status during the pre-lockdown and post-lockdown periods was found. (v) A significantly higher prevalence of 25 (OH) D deficiency was found in Saudi outpatients versus non-Saudi outpatients for all periods. (vi) Nationality was also associated with the 25 (OH) D concentration for all periods. (vii) A significant difference between the mean 25 (OH) values for the various age categories was found. (viii) There was an absence of a link between gender and nationality with the vitamin D serum level during the lockdown period. In the pre-lockdown period, the male outpatients had a 1.56 chance of being vitamin D sufficient. However, during the lockdown period, this chance decreased to less than 1 (0.850) and then began to increase to 1. (x) Age affected the 25 (OH) D serum levels positively and significantly regardless of the pandemic periods. Being male had a positive impact on 25 (OH) vitamin D levels during the pre-lockdown period only.

The population in our study may have had access to enough dietary sources of vitamin D, even during the strict lockdown. Additionally, even prior to the lockdown, sun exposure was relatively lower in our population, particularly among females, which could partly explain the generally higher prevalence of vitamin D insufficiency. This could also explain why there was no significant decline in vitamin D levels recorded.

In the present study, the prevalence of 25 (OH) D deficiency was higher among the youngest population in the lockdown and post-lockdown periods; this might be related to the dramatic change in lifestyle and limited sun exposure [41]. Surprisingly, the older group of outpatients was less likely to be affected by the consequent home confinement restrictions; this seems paradoxical since the younger population is more active and engaged in outdoor activities and more exposed to sunlight. In accordance with a recent study, we suggest that due to the restriction of person-to-person appointments, self-medication among the older population was significantly increased. In particular, vitamin D was described in the literature as one of the most commonly used drugs by the elderly population during the pandemic [42,43]. However, this needs to be thoroughly investigated.

For the younger population, the findings may be explained by the lack of regular vitamin D supplementation, reduced intake of dietary products fortified with vitamin D, the poor dietary intake and fish-enriched diet (salmon) and social habit changes, such as a preference for fast food over cooked food enriched with vitamin D. Our results further support the recent research findings, confirming that pandemic-related restrictions caused significant decreases in the vitamin D levels of school-aged children and adolescents. We also suggest that both age groups should be provided vitamin D supplementation to maintain sufficient levels of vitamin D during the home confinement restrictions [44].

In line with the current results regarding 25(OH)D insufficiency among the youngest population as a result of home confinement measures, a previous retrospective study conducted on a paediatric population [45] found statistically significant lower concentration values of vitamin D in children during the first year of the SARS-CoV-2 pandemic. Similarly, Yu L et al. have shown a decline in the 25(OH)D serum levels among children aged between 3 and 6 years during the COVID-19 pandemic. Both studies suggested that indoor confinement restricted the outdoor pleasure time and potentially reduced sun exposure, which resulted in the reduced skin synthesis of vitamin D. For this age group, vitamin D supplementation is needed for bone growth and other metabolic activities [46,47].

It is well documented that there are age-related changes in the vitamin D metabolism. Thus, the decline of serum 25(OH)D levels linked to the aging process might be due to the reduced skin production of vitamin D, the calcium absorption of circulated 1,25(OH)2D and renal production of 1,25(OH)2D [48]. As a preventive measure, to maintain adequate levels of 25 (OH) D, it is required that the daily intake of vitamin D and calcium be increased in an aged population [49].

Recent evidence has shown that cigarette consumption was increased during the nationwide isolation period [50]. Given the negative effects of nicotine, an alkaloid of tobacco, on the circulating level of vitamin D, this finding might explain the change in vitamin D metabolism in youth/adults in our study [51]. Similar to previous research, we found that the prevalence of vitamin D deficiency is higher among the young adult population.

Other factors beyond those mentioned above can be associated with physical activity, which was dramatically affected during the home confinement periods. It is possible to assume that the decrease in the serum concentration of 25 (OH) D in the population aged between 1 and 14 may be due to the lockdown measures, during which indoor sports centres, such as gyms, swimming pool, or schools, were closed, and screen time was increased. This population usually spent more time on outdoor activities. These results are corroborated by a previous retrospective study by [40,52]. Other studies have also reported that the increased consumption of high-sugar, high-fat diets, red meat, and sweet drinks increased significantly during the lockdown period [53,54]. The daily diet changes might also decrease vitamin D concentrations. This hypothesis needs to be confirmed [50].

This highlights the importance of vitamin D supplementation in the adult population and particularly for the youngest population aged between 1 and 14.

In accordance with previous studies showing that female gender is associated with vitamin D deficiency, we found that gender significantly affected the prevalence of 25 (OH) vitamin deficiency during the pre-lockdown and post-lockdown periods [55,56]. The higher prevalence of vitamin D deficiency among females might be explained by clothing practice and cultural habits, which are influenced by hot weather and lifestyle [56,57]. Despite the fact that data on clothing practices were not collected in this study, we believe that Saudi male and female dressing practices may have affected the vitamin D levels (such as wearing an abaya and a black veil, which imply covering most skin). UVR only partially influences 25 (OH) Vitamin D levels. These factors are likely to influence the amount of sun exposure regardless of the time spent outdoors. Women are more likely than men to cover their limbs and heads in public; hence, sun avoidance is more common in women. 

Clothing has been extensively mentioned as a factor contributing to vitamin D deficiency in women in the Middle East and South Asia [58,59,60].

Although they live in the same area and may have a similar diet, the fact that the prevalence of 25 (OH) D deficiency among Saudis is significantly higher than non- Saudi seems to be consistent with other previous research findings [27,61]. Factors other than sunlight exposure and diet may influence the circulating level of 25 (OH) D. Indeed, factors found to influence 25-(OH) D levels have been explored in several studies and suggest a strong genetic influence on its serum levels in various groups of patients [62,63]. Moreover, Sadat Ali et al. reported that patients who have significantly lower levels of 25 (OH) D compared to those with normal levels have the GG allele of the three SNPs VDR rs2228570, CYP2R1 rs10741657 and GC rs4588 [64].

The variation in plasma levels of 25 (OH) D in the body depends on other factors that were not investigated in this study, such as vitamin D hydroxylase, vitamin D binding protein (group-specific component) and the inactivation by cytochrome P450 CYP24 (or 25 (OH)D-24-hydroxylase) and CYP3A4. This may be one of the factors leading to vitamin D variations among different populations [65,66].

Taken together, this study provided an insight into the incidence of vitamin D insufficiency and deficiency in the Saudi population during the pandemic period, which is multifactorial. It includes, age gender, clothing practices, cultural behaviours, a high skin melanin content, vitamin D supplementation, sunlight exposure and the polymorphism of vitamin D receptors [56,67,68]. Further investigations are required to highlight the involvement of genetic variations underlying the decreased concentrations of 25 (OH) D among Saudi populations.

Age, similar to gender, obesity and other factors, is an important influence on the circulating levels of vitamin D. In general, the elderly population is more susceptible to vitamin D deficiency due to a variety of risk factors, including reduced skin vitamin D photosynthesis in response to UV, decreased sunlight exposure, decreased dietary intake, decreased skin thickness, impaired intestinal absorption, and decreased hydroxylation in the kidneys and liver [69,70].

It is also worth noting that access to food and medication was not restricted during the restricted home confinement, and home delivery was maintained. Many medical centres have shifted from person-to-person appointments to online services and arranging drug prescription during the COVID-19 lockdown. Internet access and online services can assist in ensuring that people continue to acquire vitamin D supplements and fortified dietary products during pandemic-related confinements. Altogether, this may explain some variations in vitamin D status.

### Strengths and Limitation of the Study

Despite the large sample size, which represents a major strength of our research, it should be acknowledged that our study has some limitations. This includes the lack of comprehensive patient clinical data and the possibility of vitamin D supplementation.

Furthermore, the generalizability of our results might only apply to populations with comparable food/vitamin D intakes and sun exposure indices. The measurement of the serum level of 25(OH)D in the same patients at multiple time points and the lack of data regarding vitamin D supplementation and eating habits, as well as the manner of dressing and the physical activity practices among the non-Saudi population, should also be considered. This may elucidate the effects of these variables on the 25 (OH) D levels among the outpatients. During the lockdown period, the healthcare centre reduced its person-to person appointments, thus reducing the number of consultations. The reduced the number of outpatients visiting the polyclinic during the lockdown period, which may affect the goodness-of-fit of our analysis. For example, our regression analysis did not show a significant link between 25 (OH) D level and gender and nationality during this period, which may hide the link between 25 (OH) D levels and some variables, such as gender. There was no clinical information about the reason for 25 (OH)D testing. When interpreting the results of this study, we should keep these limitations in mind, and further research is needed to confirm and extend our findings.

## 5. Conclusions

Our study did not find a statistically significant difference in the mean serum concentrations or prevalence of vitamin D insufficiency when comparing values before, during and immediately post the COVID-19 lockdown period. However, our study did show a generally increased prevalence of vitamin D insufficiency in our sample population. Our findings showed that vitamin D status is affected by age, nationality and gender. Determining vitamin D levels in different age groups in a community and in different climates of a country is necessary and has important implications for general health. Vitamin D insufficiency presents a risk to developing deficiency. Vitamin D deficiency may be considered because of the implemented lockdown restrictions that aimed at reducing the transmission of COVID-19. Regular exposure to UVR is recommended to maintain adequate vitamin D levels and to prevent vitamin D deficiency. Furthermore, while this retrospective observational study demonstrates associations, it does not allow for definitive conclusions about causality. Access to a balanced diet due to restrictions associated with lockdowns is also a consideration. If the periods of home confinement are prolonged, vitamin D supplementation may be recommended, particularly for the younger population, given its essential role in the growth and development of their skeletal muscles as well as to those at a high risk of developing complications or deficiency [71]. Further research studies remain necessary to determine the optimal indications for vitamin D supplementation.

Our findings may be considered by stakeholders, including health professionals and dietitians, national nutrition policy makers, public health organizations, and for a targeted vitamin D supplementation approach for risk groups.

## Figures and Tables

**Table 1:** Demographics and basic characteristics of outpatients involved in this study.

		25 (OH) D Status (ng/mL)			
		Total	Sufficient	Insufficient	Deficient
		*n* (%)	≥30 ng/mL	21–29	0–20
**Age**	(1–105)	7234 (100%)	2553 (35.4%)	2440(33.8%)	2217(30.7%)
**Categories**	1–14	533 (7.4%)			
	15–18	310 (4.3%)			
	19–35	3131 (43.3%)			
	36–47	1508 (20.8%)			
	48–64	1382 (19.1%)			
	65–105	370 (5.1%)			
**Mean (age) ± SD**		34.7 ± 16.8			
**Gender**					
	**Male**	5302 (73.3%)			
	**Female**	1932 (26.7%)			
**Nationality**					
	**Saudi**	5461 (75.5%)			
	**Non-Saudi**	1773 (24.5%)			

**Table 2:** 25 (OH) vitamin D status during COVID-19 pre-lockdown, lockdown, and post-lockdown periods.

	25 (OH)D Status Frequency *n* (%)	Sufficient (≥30 ng/mL) *n* = 2553 (35.3%)	Insufficient (21–29 ng/mL) *n* = 2440 (33.8%)	Deficient (0–20 ng/mL) *n* = 2217 (30.7%)	25 (OH) D Concentration (Mean ± SD, ng/mL)
**COVID-19 Periods**	**Pre-lockdown**	965 (33.5%)	1077 (37.5%)	833 (29%)	27.6 ± 11.87
	**Lockdown**	68 (34.7%)	67 (34.2%)	61 (31.1)	27.4 ± 12.52
	**Post-lockdown**	1520 (36.7%)	1296 (31.3%)	1323 (32%)	27.7 ± 13.84
	***p*-Value**	^b^: *p* < 0.001	^b^: *p* < 0.001	^b^: *p* < 0.001	^a^: 0.729

Notes: ^b^. Differences between proportions were tested using the Chi-square test. ^a^. Differences between groups’ means of 25 (OH)D were tested using an ANOVA. Abbreviations: 25 (OH) D: serum 25-Hydroxyvitamin D; STD: standard deviation.

**Table 3:** 25 (OH) vitamin D status before, during and after lockdown periods, stratified by gender.

		25 (OH) Vitamin D Serum Concentration (ng/mL)					
			Sufficient	Insufficient	Deficient	Total	*p*-Value
**Pre-lockdown**	**Female**	% Within Gender	678 (31.1%)	793 (36.4%)	710. (32.6%)	2181 (100%)	0.0000
		% of Total	23.60%	27.60%	24.70%	75.90%	
	**Male**	% Within Gender	287 (41.4%)	284 (40.9%)	123 (17.7%)	694 (100%)	
		% of Total	10.00%	9.90%	4.30%	24.10%	
	Total	% Within Gender	965 (33.6%)	1077 (37.5%)	833 (29%)	2875 (100%)	
**Lockdown**	**Female**	% Within Gender	43 (36.1%)	42 (35.3%)	34 (28.6%)	119 (100%)	0.6300
		% of Total	21.90%	21.40%	17.30%	60.70%	
	**Male**	% Within Gender	25 (32.5%)	25 (32.5%)	27 (35.1%)	77 (100%)	
		% of Total	12.80%	12.80%	13.80%	39.30%	
	Total	% Within Gender	68 (34.7%)	67 (34.2%)	61 (31.1%)	196 (100%)	
**Post-lockdown**	**Female**	% Within Gender	1095 (36.7%)	903 (30.3%)	982 (33%)	2980 (100%)	0.0350
		% of Total	26.50%	21.80%	23.70%	72.00%	
	**Male**	% Within Gender	425 (36.7%)	393 (33.9%)	341 (29.4%)	1159 (100%)	
		% of Total	10.30%	9.50%	8.20%	28.00%	
	Total	% Within Gender	1520 (36.7%)	1296 (31.3%)	1323 (32%)	4139 (100%)	

Differences between gender were tested via Chi-square test.

**Table 4:** The comparison of 25 (OH) vitamin D means between Saudi and Non-Saudi outpatients during the three periods(Pre-lockdown, lockdown and post-lockdown).

		Nationality	N (Frequency)	Mean of 25 (OH) D Concentrations	Std. Deviation	*p*-Value
**Pre-lockdown**	25 (OH) D	Non-Saudi	632	30.01	11.32	0.000
		Saudi	2255	26.91	11.94	
**Lockdown**	25 (OH) D	Non-Saudi	69	28.58	12.85	0.319
		Saudi	127	26.70	12.35	
**Post-lockdown**	25 (OH) D	Non-Saudi	1070	29.55	13.58	0.000
		Saudi	3074	27.21	13.88	

The comparison of means of 25 (OH) vitamin D between Saudi and non-Saudi patients were tested via independent *t*-test. Abbreviations: 25 (OH) D: serum 25-Hydroxyvitamin D; STD, standard deviation.

**Table 5:** 25 (OH) vitamin D status during pre-lockdown, lockdown and post-lockdown periods, stratified by nationality.

			25 (OH) Vitamin D Status (ng/mL)					
				Sufficient	Insufficient	Deficient	Total	*p*-Value
**Pre-lockdown**	**Nationality**	**Non-Saudi**						
			% Within Nationality	270 (42.9%)	254 (40.3%)	106 (16.8%)	630 (100%)	0.000
			% of Total	9.40%	8.80%	3.70%	21.90%	
		**Saudi**	% Within Nationality	695 (31%)	823 (36.7%)	727 (32.4%)	2245 (100%)	
			% of Total	24.20%	28.60%	25.30%	78.10%	
	Total		% Within Nationality	965 (33.6%)	1077 (37.5%)	833 (29%)	2875 (100%)	
**Lockdown**	**Nationality**	**Non-Saudi**						0.000
			% Within Nationality	25 (36.2%)	25 (36.2%)	19 (27.5%)	69 (100%)	
			% of Total	12.80%	12.80%	9.70%	35.20%	
		**Saudi**						
			% Within Nationality	43 (33.9%)	42 (33.1%)	42 (33.1%)	127 (100%)	
			% of Total	21.90%	21.40%	21.40%	64.80%	
	Total		% Within Nationality	68 (34.7%)	67 (34.2%)	61 (31.1%)	196 (100%)	
**Post-lockdown**	**Nationality**	**Non-Saudi**						0.000
			% Within Nationality	451 (42.1%)	382 (35.7%)	237 (22.1%)	1070 (100%)	
			% of Total	10.90%	9.20%	5.70%	25.90%	
		**Saudi**						
			% Within Nationality	1069 (34.8%)	914 (29.8%)	1086 (35.4%)	3069 (100%)	
			% of Total	25.80%	22.10%	26.20%	74.10%	
	Total		% Within Nationality	1520 (36.7%)	1296 (31.3%)	1323 (32%)	4139 (100%)	

**Table 6:** Comparison of means of concentration of 25 (OH) D between age groups in the three periods.

		Means of 25 (OH) D Concentrations (ng/mL)		
Age Categories	N (Frequency)	Pre-Lockdown	Lockdown	Post-Lockdown
1–14	226	32.75	23.93	28.27
15–18	99	23.76	22.58	25.19
19–35	1439	26.23	27.49	26.60
36–47	501	27.35	26.52	28.18
48–64	480	28.97	28.37	29.53
65–105	142	32.03	36.65	30.52
Total	2887	27.59	27.36	27.81

**Table 7:** Linear regression analysis of the 25 (OH) D level stratified by age, gender and nationality during pre-lockdown, lockdown, and post-lockdown periods.

Coefficients ^a^									
Periods			Unstandardized Coefficients		Standardized Coefficients			95.0% Confidence Interval for B	
			B	Std. Error	Beta	t	Sig.	Lower Bound	Upper Bound
**Pre-lockdown**	1	(Constant)	28.02	0.68		40.91	0.00	26.67	29.36
		Age	0.03	0.01	0.04	2.31	0.02	0.01	0.06
		Nationality	−2.65	0.53	−0.09	−4.95	0.00	−3.70	−1.60
		Gender	2.69	0.52	0.10	5.22	0.00	1.68	3.70
**Lockdown**	1	(Constant)	24.26	2.54		9.54	0.00	19.24	29.28
		Age	0.12	0.05	0.17	2.31	0.02	0.02	0.22
		Nationality	−1.76	1.86	−0.07	−0.95	0.34	−5.44	1.91
		Gender	−1.42	1.90	−0.06	−0.75	0.45	−5.17	2.32
**Post-lockdown**	1	(Constant)	27.22	0.65		41.70	0.00	25.94	28.50
		Age	0.07	0.01	0.09	5.48	0.00	0.05	0.10
		Nationality	−2.28	0.49	−0.07	−4.62	0.00	−3.25	−1.31
		Gender	−0.93	0.49	−0.03	−1.92	0.06	−1.88	0.02

^a^ Dependent variable: total vitamin D.

**Table 8:** Regression analysis of the 25 (OH) vitamin D status stratified by gender.

			Variables in the Equation						95% Confidence Interval	
Periods			**B**	**S.E.**	**Wald**	**df**	**Sig.**	**Odd Ratio**	**Lower**	**Upper**
Prelockdown	Step 1 ^a^	Gender	0.447	0.09	24.695	1	0.000	1.563	1.311	1.864
		Constant	−0.8	0.046	296.099	1	0.000			
Lockdown	Step 1 ^a^	Gender	−0.16	0.309	0.277	1	0.599	0.850	0.463	1.558
		Constant	−0.57	0.191	8.908	1	0.003			
Post-lockdown	Step 1 ^a^	Gender	0	0.072	0.002	1	0.964	0.997	0.866	1.147
		Constant	−0.54	0.038	204.355	1	0.000			

^a^ Variable(s) entered on step 1: gender.

## Data Availability

Data are available upon request due to restrictions in privacy or ethical issues. The data presented in this study are available upon request from the corresponding author. The data are not publicly available due to confidentiality and ethical issues.
